# Gene expression profiling in the stress control brain region hypothalamic paraventricular nucleus reveals a novel gene network including Amyloid beta Precursor Protein

**DOI:** 10.1186/1471-2164-11-546

**Published:** 2010-10-08

**Authors:** Amalia Tsolakidou, Ludwig Czibere, Benno Pütz, Dietrich Trümbach, Markus Panhuysen, Jan M Deussing, Wolfgang Wurst, Inge Sillaber, Rainer Landgraf, Florian Holsboer, Theo Rein

**Affiliations:** 1 Max-Planck Institute of Psychiatry, Munich, Germany; 2 Helmholtz Centre and Technical University Munich, Institute for Developmental Genetics, Neuherberg, Germany; and German Centre for Neurodegenerative Diseases, Munich, Germany; 3 Department of Psychiatry and Psychotherapy, Technical University of Munich

## Abstract

**Background:**

The pivotal role of stress in the precipitation of psychiatric diseases such as depression is generally accepted. This study aims at the identification of genes that are directly or indirectly responding to stress. Inbred mouse strains that had been evidenced to differ in their stress response as well as in their response to antidepressant treatment were chosen for RNA profiling after stress exposure. Gene expression and regulation was determined by microarray analyses and further evaluated by bioinformatics tools including pathway and cluster analyses.

**Results:**

Forced swimming as acute stressor was applied to C57BL/6J and DBA/2J mice and resulted in sets of regulated genes in the paraventricular nucleus of the hypothalamus (PVN), 4 h or 8 h after stress. Although the expression changes between the mouse strains were quite different, they unfolded in phases over time in both strains. Our search for connections between the regulated genes resulted in potential novel signalling pathways in stress. In particular, Guanine nucleotide binding protein, alpha inhibiting 2 (GNAi2) and Amyloid β (A4) precursor protein (APP) were detected as stress-regulated genes, and together with other genes, seem to be integrated into stress-responsive pathways and gene networks in the PVN.

**Conclusions:**

This search for stress-regulated genes in the PVN revealed its impact on interesting genes (GNAi2 and APP) and a novel gene network. In particular the expression of APP in the PVN that is governing stress hormone balance, is of great interest. The reported neuroprotective role of this molecule in the CNS supports the idea that a short acute stress can elicit positive adaptational effects in the brain.

## Background

Stressful life events are among the most potent factors that can trigger the development of psychiatric disorders such as depression and anxiety disorders [1-3]. Aberrations in the function of the hypothalamus-pituitary-adrenal (HPA) axis, the key control system of the body to balance stress hormones and the response to stress, already exist prior to the onset of clinical symptoms [4]. The functionality of the HPA axis is mainly governed by genetic endowment, but developmental influences and life events, in particular stress experience early in life, can re-program the settings of the HPA axis [5-8].

The hypothalamus, as part of the HPA axis, is the centre of stress response and a region of the brain that integrates different stress signalling neuronal pathways. The hypothalamic paraventricular nucleus (PVN) is the main area of the hypothalamus where the corticotropin-releasing hormone (CRH, also known as corticotropin-releasing factor, CRF), the crucial neuropeptide that activates the secretion of corticotropin (ACTH), is produced and released. This effect, in turn, causes the secretion of glucocorticoids from the adrenal glands [9,10]. The levels of ACTH and glucocorticoids in the plasma can be used as markers to monitor stress levels [11]. In addition to CRH, other hormonal molecules such as arginine vasopressin (AVP) and oxytocin contribute to the regulation of the HPA axis' activity [12-16].

In major depression a hypothalamic hyper-drive is observed. This is constituted by the elevation of CRH, AVP and oxytocin, which may influence the clinical symptoms. In the PVN of depressed patients the total number of CRH expressing neurons showing co-localisation with AVP and the amount of CRH-mRNA are increased [17-19]. An important hallmark of HPA axis regulation is the negative feedback exerted by glucocorticoids on the production and release of CRH and AVP in the PVN, as well as of ACTH in the pituitary. Substantial evidence has been provided that this attenuation activity, which is an integral part of the stress response, is impaired in patients suffering from major depression [20].

Recently, we have determined stress-regulated genes in the hippocampus, a higher limbic centre in the brain, of two inbred mouse strains with differential psycho-phenotype, namely C57BL/6J and the DBA/2J by employing microarray analysis [21]. The choice of these mouse strains is based on reports that these strains have differential response to stress [22,23], differential basal anxiety [24] and differ in both, their cognitive abilities [25] and sensitivity to antidepressants [26,27]. The microarray method, that belongs to the chip-based whole genome technologies, allows for unbiased approaches with the potential to identify new candidate genes and gene networks [20]. Our results had led to the successful identification of stress-regulated genes and suggestion of possible signal transduction pathways involved.

As it is of great interest to study the impact of stress in brain regions directly involved in stress regulation, in this parallel study we have focused on the impact of stress experience on hypothalamic PVN governing the stress response. The PVN area was micropunctured from the brains of C57BL/6J and DBA/2J mice that had been stressed once by forced swimming [21] and mRNA profiles were determined by microarray analysis. Forced swimming is also being used routinely as test to monitor depression-like behaviour and drug effects [28]. We report that Guanine nucleotide binding protein, alpha inhibiting 2 and Amyloid β (A4) precursor protein (APP) are up-regulated after stress and we suggest a novel gene network involved in stress response in the two mouse strains. This network implies that the expression of APP might be a neuroprotective component of stress adaptation in the PVN.

## Results

### Basal gene expression differences between C57BL/6J and DBA/2J mice in PVN

**Figure 1:**
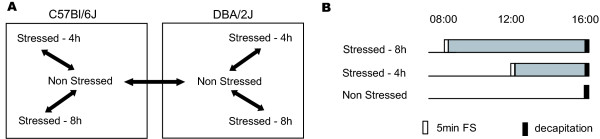
**Schematic outline of the expression comparisons and stress experiments**. A. Samples of non-stressed animals were compared to those of stressed animals (sacrificed either 4 h or 8 h later). Two mouse strains were used (C57BL/6J and DBA/2J). A comparison of basal expression was also included. B. C57BL/6J and DBA/2J mice were subjected to 5 min forced swimming (FS) either at 8:00 or at 12:00, and sacrificed 4 h (Stressed-4 h) or 8 h (Stressed-8 h) later at 16:00. Control mice were sacrificed at 16:00 without having been stressed (Non Stressed).

To compare expression profiles between the two mouse strains C57BL/6J and DBA/2J and evaluate expression changes at different time points after stress exposure, we used cDNA microarrays. We first evaluated the differences of the two mouse strains in their basal expression profile in the PVN (see Fig. 1A for scheme of all comparisons). The microarray analysis revealed 670 genes with more than 1.4 fold difference in expression.

Among the most pronounced expression differences we identified genes with a variety of function, such as protein kinase activity, genes with extracellular ligand-gated ion channel activity, genes involved in protein homeostasis (e.g. ubiquitin-dependent protein catabolism, protein biosynthesis-, ribosome-, ribonucleoprotein complex-related genes), cell surface (defence response, peptide antigen binding, antigen presentation, endogenous peptide antigen), and chromosomal processes (chromosome segregation, response to DNA damage stimuli, mitosis, DNA repair, DNA metabolism, homologous chromosome segregation, cell cycle, cell division); examples are provided in table 1 (for the whole list see Additional File 1).

**Table 1:** Differentially expressed genes in the PVN between mouse strains (examples, +: higher, -: lower expressed in DBA/2J vs C57BL/6J)

GenBank ID	Gene symbol	Gene name	Fold regulation
AI845809	D10Bwg1379e	DNA segment, Chr 10, Brigham & Women's Genetics 1379 expressed	-5.22

AI666719	Luc7l2	LUC7-like 2 (S. cerevisiae)	-3.11

AA057995	Mrps18c	Mitochondrial ribosomal protein S18C	-3.07

AI893657	Map3k12	Mitogen activated protein kinase kinase kinase 12	-3.01

AI836990	Pttg1	Pituitary tumor-transforming 1	-2.93

AI661130	H2-Q1	Histocompatibility 2, Q region locus 1	-2.88

AI850098	Glul	Glutamate-ammonia ligase (glutamine synthase)	-2.83

AI428471	Spock3	Sparc/osteonectin, cwcv and kazal-like domains proteoglycan 3	-2.74

AI834783	Gabra1	Gamma-aminobutyric acid (GABA-A) receptor, subunit alpha 1	-2.04

AI842005	App	Amyloid beta (A4) precursor protein	1.44

AI846532	Gnao1	Guanine nucleotide binding protein, alpha o	2.19

AI838288	Kcnj9	Potassium inwardly-rectifying channel, subfamily J, member 9	2.7

AI852059	Usp46	RIKEN cDNA 1700112M01 gene	3

### ACTH and gene expression profiles after forced swimming

**Figure 2:**
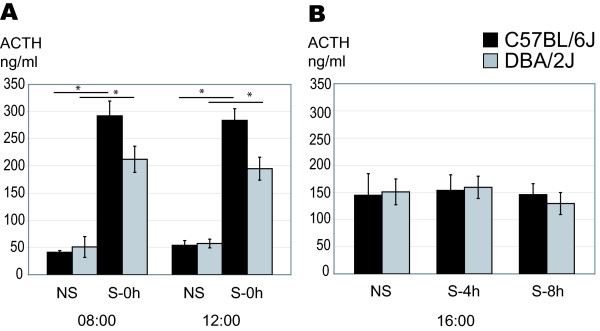
**Plasma ACTH concentrations of stressed and non stressed mice**. A. To monitor the ACTH stress response, a group of mice was stressed (S-0 h) and sacrificed immediately thereafter (at 8:00 or at 12:00) in parallel to a control non stressed group (NS). 5 min forced swimming caused an instantaneous induction of ACTH plasma levels in C57BL/6J and DBA/2J mice either at 8:00 or at 12:00; n = 5 for each condition. Data represent mean and SD. B. Plasma ACTH levels of non-stressed (NS) and stressed animals at the time of sacrifice i.e. 4 h and 8 h after stress (S-4 h, S-8 h) were determined and show no significant difference between groups; n = 10 for each condition. Data represent mean and SD. Brains of these animals were saved for microarray analysis.

To determine transcriptional profiles after stress, mice had been subjected to forced swimming for 5 min at 8:00 h or 12:00 h and then decapitated at 16:00 h in order to isolate the brains for our microarray studies as previously described [21] (experimental plan depicted in Fig. 1B). In this previous study the impact of forced swim stress on stimulation of the HPA axis was evaluated by the determination of corticosterone (CORT) levels in the serum. In order to monitor the activation of the pituitary that responds to the CRF release, we also measured the ACTH levels. A set of animals of both strains was decapitated directly after stress exposure at 08:00 h and at 12:00 h (non stressed control animals were also included). The results show a clear increase for ACTH after stress in both mouse strains and at both time points selected in our study (Fig. 2), like previously also for CORT [21]. Thus, the pituitary as well as the adrenals responded immediately to the stressor. When animals were decapitated 4 h or 8 h after the stress, the levels of ACTH were not different from the non stressed controls, indicative of a functional negative feedback mechanism (Fig. 2). The levels in non-stressed and stressed animals were higher at 16:00 than in the morning though, most likely due to the circadian rhythm of the mice.

To reveal gene expression differences, samples from non stressed mice were compared to samples from mice that had been stressed 4 h or 8 h before decapitation. In C57BL/6J mice, 123 genes were ≥1.4 fold regulated 4 h after stress and 88 genes 8 h after stress. In DBA/2J mice, 185 genes and 96 genes were regulated at the respective time points. (Additional File 2, 3, 4 and 5).

Examples of the most interesting regulated genes are highlighted in tables 2, 3, 4, 5: In C57BL/6J mice, among the genes regulated 4 h after stress we distinguish phosphodiesterase 1C (PDE1C), Mitogen-activated protein kinase kinase kinase kinase 3 (MAP4K3) and polymerase (DNA directed) delta 1, catalytic subunit (POLD1) (see Table 2). In the group of genes regulated 8 h after stress we noticed heat shock protein 1, alpha (Hspca), Guanine nucleotide binding protein, alpha o (GNAO1) and the regulator of G-protein signaling 2 (RGS2) (see Table 3).

**Table 2:** Examples of genes that are up- or down-regulated 4 h after stress in C57BL/6J mice

GenBank ID	Gene symbol	Gene name	Fold regulation
AI255458	Ambp	Alpha 1 microglobulin/bikunin	-1.98

AI842824	Pstk	Phosphoseryl-tRNA kinase	-1.66

AI853074	Pde1c	Phosphodiesterase 1C	-1.5

AI853855	Ndufa8	NADH dehydrogenase (ubiquinone) 1 alpha subcomplex, 8	-1.45

AI836325	Pak2	P21 (CDKN1A)-activated kinase 2	-1.44

AI844849	Cadps	Ca < 2+ > dependent activator protein for secretion	1.42

AI427715	Map4k3	Mitogen-activated protein kinase kinase kinase kinase 3	1.43

AI847671	Cab39	Calcium binding protein 39	1.52

AI836406	Ubqln2	Ubiquilin 2	1.57

AI465497	Dpysl2	Dihydropyrimidinase-like 2	1.58

AI851668	Pold1	Polymerase (DNA directed), delta 1, catalytic subunit	1.65

AI848676	Ubl3	Ubiquitin-like 3	1.7

AI848690	Cacna1a	Calcium channel, voltage-dependent, P/Q type, alpha 1A subunit	1.76

AA433755	Bag4	BCL2-associated athanogene 4	2.03

AI845595	Ogfrl1	Opioid growth factor receptor-like 1	2.59

**Table 3:** Examples of Genes up- or down-regulated 8 h after stress in C57BL/6J mice

GenBank ID	Gene symbol	Gene name	Fold regulation
AI661341	Adam10	A disintegrin and metalloprotease domain 10	-2.66

AI852787	Insig1	Insulin induced gene 1	-2.56

AK049469	Baiap2	Brain-specific angiogenesis inhibitor 1-associated protein 2	-2.34

AI666719	Luc7l2	LUC7-like 2 (S. cerevisiae)	-2.16

AI841344	Hspca	Heat shock protein 1, alpha	-1.97

AI414590	Srpk2	Serine/arginine-rich protein specific kinase 2	-1.91

AI838689	Ptp4a2	Protein tyrosine phosphatase 4a2	-1.69

AI841196	Gja12	Gap junction membrane channel protein alpha 12	-1.59

AI848233	Spry2	Sprouty homolog 2 (Drosophila)	-1.58

AI847890	Plp1	Proteolipid protein (myelin) 1	-1.42

AI854312	Kif3b	Kinesin family member 3B	1.45

AI841804	Nme1	Expressed in non-metastatic cells 1, protein	1.47

AI846532	Gnao1	Guanine nucleotide binding protein, alpha o	1.51

AI324856	Idh3b	Isocitrate dehydrogenase 3 (NAD+) beta	1.53

AI839703	Scn4b	Sodium channel, type IV, beta	1.57

AI847923	Rgs2	Regulator of G-protein signaling 2	1.61

AI837318	Abcf2	ATP-binding cassette, sub-family F (GCN20), member 2	1.77

**Table 4:** Examples of Genes up- or down-regulated 4 h after stress in DBA/2J mice

GenBank ID	Gene symbol	Gene name	Fold regulation
10357	Hhex	Hematopoietically expressed homeobox	-2.05

13913	Ppp3cb	Protein phosphatase 3, catalytic subunit, beta isoform	-1.84

7804	Pbef1	Pre-B-cell colony-enhancing factor 1	-1.65

5652	Nr3c1	Nuclear receptor subfamily 3, group C, member 1	-1.55

6999	Rps6	Ribosomal protein S6	-1.52

10119	Bmp6	Bone morphogenetic protein 6	-1.44

4373	Prkcz	Protein kinase C, zeta	1.71

18990	Trh	Thyrotropin releasing hormone	1.75

1377	Ina	Internexin neuronal intermediate filament protein, alpha	1.84

19152	Nfatc1	Nuclear factor of activated T-cells, cytoplasmic, calcineurin-dependent 1	1.92

9897	Gnai2	Guanine nucleotide binding protein, alpha inhibiting 2	2.11

349	Mapk3	Mitogen activated protein kinase 3	2.15

2405	Nmt1	N-myristoyltransferase 1	2.29

16726	Vamp2	Vesicle-associated membrane protein 2	2.43

12438	Map1lc3a	Microtubule-associated protein 1 light chain 3 alpha	2.47

**Table 5:** Examples of Genes up- or down-regulated 8 h after stress in DBA/2J mice

GenBank ID	Gene symbol	Gene name	Fold regulation
AI327089	Homer3	Homer homolog 3 (Drosophila)	-2.26

AI465481	Za20d2	Zinc finger, A20 domain containing 2	-2.07

AA617397	Chd4	Chromodomain helicase DNA binding protein 4	-2.02

AA024364	Slc6a12	Solute carrier family 6 (neurotransmitter transporter, betaine/GABA), member 12	-1.85

AA038810	Prc1	Protein regulator of cytokinesis 1	-1.72

AA266744	Tlr7	Toll-like receptor 7	-1.64

AI449276	Ctso	Cathepsin O	-1.49

AI854447	Set	SET translocation	1.42

AI451561	Tcf7l2	Transcription factor 7-like 2, T-cell specific, HMG-box	1.47

BE283555	Hspa1a	Heat shock protein 1A	1.54

W10526	Cacng1	Calcium channel, voltage-dependent, gamma subunit 1	1.56

AI447986	Nfia	Nuclear factor I/A	1.59

AI845883	Tebp	Telomerase binding protein, p23	1.61

AI843768	App	Amyloid beta (A4) precursor protein	1.64

AI843786	Cdkn1b	Cyclin-dependent kinase inhibitor 1B (P27)	1.66

AI661341	Adam10	A disintegrin and metalloprotease domain 10	1.53

AI836959	Adam10	A disintegrin and metalloprotease domain 10	1.43

In DBA/2J mice, among the genes regulated 4 h after stress, mitogen activated protein kinase 3 (MAPK3), Guanine nucleotide binding protein, alpha inhibiting 2 (GNAi2), nuclear factor of activated T-cells, cytoplasmic, calcineurin-dependent 1 (NFATC1) were most striking (Table 4), while among the genes regulated 8 h after stress Amyloid beta (A4) precursor protein (APP), cyclin-dependent kinase inhibitor 1B (P27) (CDKN1B), Transcription factor 7-like 2, T-cell specific, HMG-box (TCF7L2) were most prominent (Table 5).

### The reaction to stress on the transcriptome level differs between mouse strains and displays phases

To gain more insight into the potential functions of the regulated genes, each set of regulated genes was sorted according to their ontology. Genes coding for mitochondrial, biosynthetic and metabolic molecules, receptors, signal transduction molecules, transcription and mRNA processing molecules, ion channels and ion transport molecules, vesicular transport molecules and cytoskeleton components are highly represented (Table 6). While the proportion of receptors and signal transduction molecules decreased between 4 h and 8 h after stress, the proportion of mitochondrial, biosynthetic and metabolic proteins increased in both mouse strains.

**Table 6:** Ontologically sorted genes regulated 4 h and 8 h after stress

Ontology	C57BL/6 Stress-4 h	C57BL/6 Stress-8 h	DBA/2J Stress-4 h	DBA/2J Stress-8 h
mitochondrial, biosynthetic and metabolic proteins	**16%**	**20%**	**24%**	**33%**

vesicular transport and components of cytoskeleton	14%	8%	21%	9%

receptors and signal transduction molecules	14%	19%	11%	13%

transcription and mRNA molecules	8%	11%	17%	13%

ion channels and ion transport	9%	17%	3%	6%

molecules with other function	39%	25%	24%	26%

These observations suggested that the main focus of expression regulation after stress is shifted over time. To further address the question of the durability of the gene expression changes, we compared the genes regulated at 4 h with those regulated at 8 h after stress for each mouse strain. Surprisingly, the set of regulated genes completely changed from 4 h to 8 h after stress in both mouse strains (Table 7). Genes that responded to the stressor after 4 h showed normalized expression levels (i.e. less than 1.4 fold regulation) after 8 h, while other genes showed up as regulated at that time (Table 7). We also observed that the stress-induced changes of the PVN transcriptome were entirely different between C57BL/6J and DBA/2J mice. Interestingly though, a convergence was apparent 8 h after stress (Table 7).

**Table 7:** Number of genes regulated 4 h and 8 h after stress in C57BL/6J or DBA/2J

	Stressed - 4 h	*common S4 h - S8 h*	Stressed - 8 h
**C57BL/6**	123	1	88

***common between C57BL/6 and DBA/2J***	0		7

**DBA/2J**	185	0	96

### Possible signalling pathways elicited after forced swimming in the PVN

**Figure 3:**
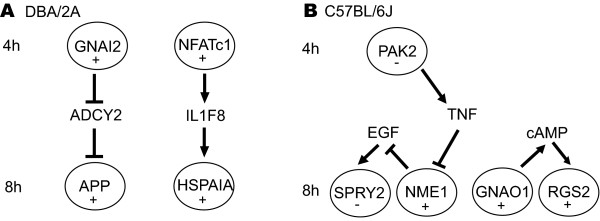
**Stress-induced pathways in the PVN of the hypothalamus of DBA/2J (A) and C57BL/6 (B) mice**. Using a pathway building program, connections between the group of genes regulated at 4 h and the group of genes regulated at 8 h after stress were determined, as well as connections within groups. Gene names identified on the microarray are circled, the direction of expression change is indicated by plus or minus signs. GNAi2: guanine nucleotide binding protein, alpha inhibiting 2; NFATc1: nuclear factor of activated T-cells, cytoplasmic, calcineurin-dependent 1; APP: amyloid beta (A4) precursor protein; HSPAIA: HSP70 heat Shock protein 70; ADCY2: adenyl cyclase 2;IL1F8: Interleukin 18; PAK2: p21 (CDKN1A) activated kinase 2); SPRY2: sprouty homolog 2 (Drosophila); NME1: expressed in non-metastatic cells 1 protein; GNAO1: guanine nucleotide binding protein, alpha; RGS2: regulator of G-protein signaling 2; TNF: tumor necrosis factor alpha; EGF: epidermal growth factor (beta-urogastrone); cAMP: cyclic AMP. Arrows indicate a positive, blunted lines a negative effect on expression, molecular synthesis or activity of the respective entity (e.g. protein, small molecule etc.).

The observation that the percentage of receptors and signalling molecules among the regulated genes decreased from 4 h to 8 h after stress (Table 6), together with the phased reaction of the transcriptome to stress (Table 7), led us to hypothesise that genes regulated at 4 h have pathway connections to genes regulated at 8 h. Employing a pathway building program to test this hypothesis, we identified links between genes responding at 4 h and genes responding at 8 h. For example, GNAi2, found to be up-regulated 4 h after stress in DBA/2J mice, is upstream of APP, which is up-regulated 8 h after stress. Additionally, NFATC1, found to be up-regulated 4 h after stress, is upstream of heat shock protein 70, HSPAIA (HSP70), which increases 8 h after stress (Fig. 3A).

In C57BL/6J mice, p21[CDKN1A]-activated kinase 2 (PAK2), down-regulated 4 h-after stress, inhibits the expression of 'expressed in non-metastatic cells 1 protein' (NME1) via regulation of tumor necrosis factor alpha (TNFα, FIG. 3B). NME1, in turn, is upstream of sprouty homolog 2 (Drosophila; SPRY2), which is regulated 8 h after stress. Another connection between genes that are both regulated 8 h after stress is between GNAO1 and RGS2 (Fig 3B).

### Validation of GNAi2 and APP expression and regulation in the PVN

**Figure 4:**
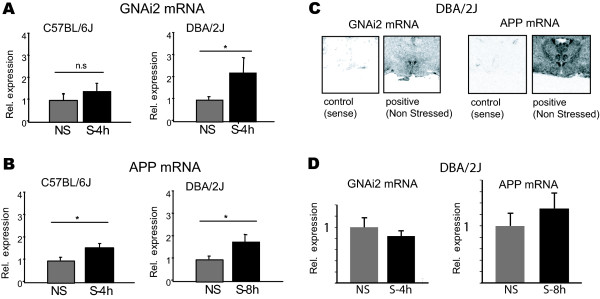
**Increased expression of GNAi2 and APP in the PVN of the hypothalamus of stressed DBA/2J mice**. A. Real-time PCR analysis shows an increase of GNAi2 mRNA in the PVN region of the hypothalamus of DBA/2J mice 4 h after stress. B. Real-time PCR analysis shows an increase in APP mRNA in the PVN 8 h after stress. C. Region-specific expression of GNAi2 and APP in the PVN of the hypothalamus of DBA/2J mice as shown by *in situ *hybridization. D. Real-time PCR analysis revealed no significant change of GNAi2 and APP in combined RNA samples from the hypothalamic region just anterior and posterior of the PVN (medial preoptic nucleus, partly from the dorsomedial hypothalamic nucleus and the periventricular hypothalamic nucleus).

To validate and further analyse the expression changes of the genes GNAi2 and APP that are linked by a pathway in the PVN of DBA/2J mice, we used real-time PCR. RNA samples from the original punctures were amplified (one round of amplification) and subjected to RT-PCR without pooling. The results confirmed the up-regulation of GNAi2 4 h after stress (2.2 fold, Fig. 4A) detected by the microarray (2.1 fold). To test whether this regulation is specific for DBA/2J mice, or may also occur in C57BL/6J mice (but was not detected in our microarray screening, because of the selection filtering), we also tested the respective samples from C57BL/6J mice. The results showed a non-significant increase in this mouse strain, implying that the regulation is rather strain-specific (Fig. 4A).

Similarly to GNAi2, we validated the expression and regulation of APP 8 h after forced swimming by real-time PCR (Fig. 4B), which was found in the microarray analysis.

To visualize the regulation of this expression with spatial resolution in the PVN, *in situ *hybridization was performed on coronal brain-sections, followed by semi-quantification of the mRNA signal. The results showed a strong signal in the PVN area (Fig. 4C) and the analysis confirmed again the up-regulation to a level of 1.6 fold (data not shown). *In situ *hybridization showed also specific expression of APP in the PVN (Fig. 4C).

Finally, to test whether stress-regulation of GNAi2 and APP is specific to the PVN, we also analysed the hypothalamic region just anterior and posterior to the PVN. The results show no significant change in the expression levels of GNAi2 and APP for the respective time point (Fig. 4D).

### Clustering analysis of GNAi2 - APP

Having corroborated a potential role of the GNAi2 - APP connection in stress response of DBA mice, we used clustering analysis to identify genes that display similar expression changes throughout the conditions we analysed. In setting up the clustering analysis, we considered both up- and down-regulation, because some transcriptional regulators, such as GR, are able to both up- and down-regulate genes. Genes identified in a clustering analysis may be under a common transcriptional control, or influencing each other.

**Figure 5:**
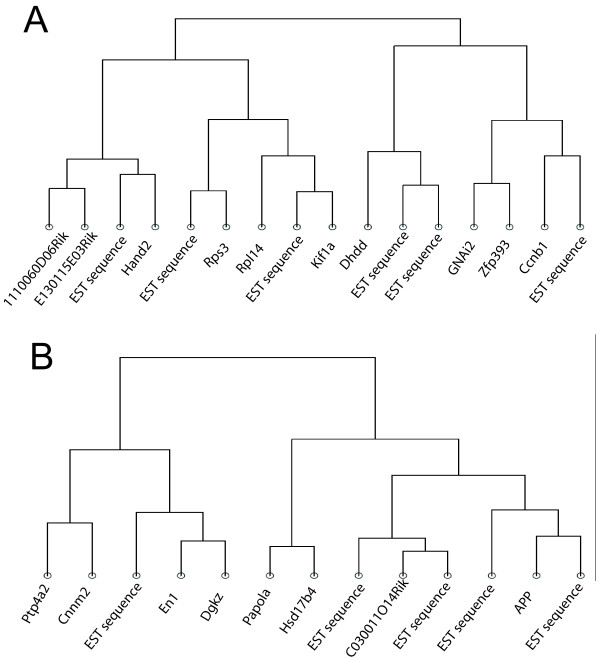
**Dendrograms of genes that display expression changes similar to GNAi2 and APP throughout the analysed conditions**. A. Neighbouring transcripts for GNAi2. Displayed are neighbours of one of the two spots on the microarray. The genes were 1110060D06Rik: RIKEN cDNA 1110060D06 gene, E130115E03Rik: RIKEN cDNA E130115E03 gene, HAND2: heart and neural crest derivatives expressed transcript 2, RPS3: ribosomal protein S3, RPL14: ribosomal protein L14, KIF1A: kinesin family member 1A, DHDDS: dehydrodolichyl diphosphate synthase, ZFP393: zinc finger protein 393, CCNB1: cyclin B1, related sequence 1 and several EST sequences as well as additional transcripts for GNAi2 from the second spotted transcript (C030044B11Rik: RIKEN cDNA C030044B11 gene, SLC2A5: solute carrier family 2 (facilitated glucose transporter), member 5, COPS5: COP9 (constitutive photomorphogenic) homolog, subunit 5 (Arabidopsis thaliana), MAP3K12: mitogen activated protein kinase kinase kinase 12, AI848100: Expressed sequence AI848100, MRPL12: mitochondrial ribosomal protein L12, SLFN2: schlafen 2, 2810046L04Rik: RIKEN cDNA 2810046L04 gene, KAZALD1: Kazal-type serine protease inhibitor domain 1. B. Neighbouring genes for APP. PTP4A2: protein tyrosine phosphatase 4a2, CNNM2: cyclin M2, EN1: engrailed 1, PAPOLA: poly (A) polymerase alpha, HSD17B4: hydroxysteroid (17-beta) dehydrogenase 4, C030011O14Rik: RIKEN cDNA C030011O14 gene and several EST sequences.

The dendrograms revealed 10 and 15 genes for GNAi2 (two transcripts of this gene were spotted) and 12 for APP (Fig. 5, see also Additional File 6 for the expression profiles). Several genes with known function were among these nearest neighbours.

**Figure 6:**
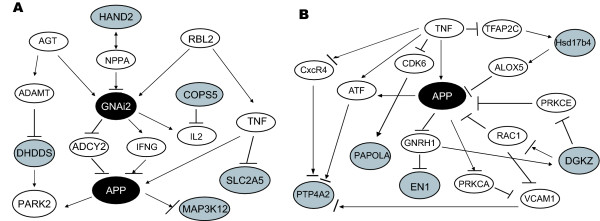
**Signalling pathways linking genes whose expression profile clusters with the expression profile of GNAi2 (A) or APP (B)**. A. Genes resulting from the clustering analysis are in grey ellipses (**HAND2**: heart and neural crest derivatives expressed 2, **DHDDS**: dehydrodolichyl diphosphate synthase, **COPS5**: COP9 constitutive photomorphogenic homolog subunit 5 (Arabidopsis), **SLC2A5**: solute carrier family 2 (facilitated glucose/fructose transporter), member 5, **MAP3K12**: mitogen-activated protein kinase kinase kinase 12) and intermediate molecules are in open ellipses (AGT: angiotensinogen (serpin peptidase inhibitor, clade A, member 8), ADAMTSL1: similar to ADAMTS-like protein 3 precursor (ADAMTSL-3) (Punctin-2), PARK2: parkinson disease (autosomal recessive, juvenile) 2, parkin, NPPA: natriuretic peptide precursor A, IFNG: interferon, gamma, RBL2 retinoblastoma-like 2 (p130), IL2: interleukin 2, TNF: tumor necrosis factor (TNF superfamily, member 2). B. Genes resulting from the clustering analysis are in grey ellipses (**HSD17B4**: hydroxysteroid (17-beta) dehydrogenase 4**, DGKZ**: diacylglycerol kinase, zeta 104 kDa, **EN1**: engrailed homeobox 1**, PAPOLA**: poly(A) polymerase alpha), **PTP4A2**: protein tyrosine phosphatase 4a2) and intermediate molecules are in open ellipses (CDK6: cyclin-dependent kinase 6, ALOX5: arachidonate 5-lipoxygenase, PRKCE: protein kinase C, epsilon, RAC1: ras-related C3 botulinum toxin substrate 1 (rho family, small GTP binding protein Rac1) PRKCA: protein kinase C, alpha, GNRH1: gonadotropin-releasing hormone 1 (luteinizing-releasing hormone), ATF: activating transcription factor, CXCR4: chemokine (C-X-C motif) receptor 4, VCAM1: vascular cell adhesion molecule 1)

To test potential, already described connections between these genes, we again used a pathway building program. Interestingly, we found that GNAi2 is located in a signalling cascade, where 'Heart and neural crest derivatives expressed transcript 2' (HAND2) is upstream, APP is downstream and MAP3K2 further downstream. DHDDS and GNAi2 share a common upstream regulator (AGT) as well as a common downstream target (PARK2). GNAi2 and COPS5 have a common target as well. In addition, GNAi2 and APP are found both downstream of another common regulator (RBL2, Fig. 6A). Furthermore, for APP we identified common upstream regulators with PAPOLA (CDC2 and TNF), EN1 as its regulator and HSD17B4 as its target. Interestingly, DGKZ and APP are linked in a feedback loop (Fig. 6B). Thus, the clustering analysis revealed functionally related genes, indeed.

## Discussion

The PVN of the hypothalamus is pivotal in governing physiological stress response. We examined the impact of forced swimming as an acute stressor on gene expression in the PVN of C57BL/6 and DBA/2J mice. These inbred mouse strains have been used as a genetic animal model of depression-like behaviour and are characterized by a different stress-responsiveness, since DBA/2J mice display a stronger behavioural response to stressful conditions [22,23,29]. We discovered that the stress-regulated genes code mainly for receptors and signal transduction molecules, as well as numerous biosynthetic molecules. This result is consistent with a previous study of gene expression profiling in the hypothalamus of mice stressed by immobilization, where genes involved in energy and lipid metabolism, apoptosis, signal transduction, DNA repair, protein biosynthesis, and structure integrity-related genes were found. However, this study referred to the entire hypothalamus and the number of genes studied was comparatively small (6016 genes) [30], while in our study approximately 19900 gene transcripts have been studied. In addition, one should keep in mind that categorically distinct acute stressors elicit distinct transcriptional profiles in the PVN [31]. Nevertheless, a few genes were found in both studies, for example ATP synthase, H^+ ^transporting mitochondrial F1 complex and ribosomal protein L30 [30].

In principle, all genes that we found differentially expressed or differentially regulated between the two mouse strains are candidates for the explanation of the differential response to some stressors, reflecting the previously proposed differences in corticosteroid signalling [32]. In addition, we checked genes with known functions that could contribute to the strain differences such as POMC1 (Pro-opiomelanocortin-alpha), GR (Glucocorticoid receptor), CRHR1 and CRHR2 (Corticotrophin Releasing Hormone Receptor 1 and 2). Only GR showed differential regulation, namely a down-regulation in DBA/2J mice 4 h after stress. Causality is difficult to prove in feedback systems such as the HPA axis, i.e. when considering the complex connections of compensatory mechanisms that emerged in this evolutionarily old threat response system [33]. Nevertheless, this difference could be indicative of differential stress signalling, as GR is the most important mediator of corticosteroid action.

Since DBA/2J mice also exhibit a higher sensitivity to antidepressants than C57BL/6 [34] we also specifically investigated genes that had been associated with antidepressant response before, such as the immunophilin FKBP5 [35], which is an efficient regulator of GR [36], the multidrug resistance protein ABCB1A/B that determines brain tissue penetration of many antidepressant drugs [37], the serotonin receptor 5 hT2A, and the transporter proteins SLC6A4 and SLC6A15 (soluble carrier family 6 member 4 and 15) [38]. Among those, SLC6A15 was down-regulated 4 h after stress in DBA/2J mice, but not in C57BL/6 mice. Since stress and GR action is intertwined with the action of antidepressants [3,39,40], also any of the stress-induced genes could contribute to the action of antidepressants. Like with other screening studies, it is certainly premature to delineate direct candidates for novel antidepressant targets or for diagnostic markers from this study. However, the synopsis of our results together with results from different screening efforts in genetics [38], proteomics, metabolomics etc., will yield convergence and thus allow selection of the most promising candidates.

Our study provides strong evidence for a time-phased response of the PVN transcriptome to the stressor. We have previously described a phased stress response for the CA3 hippocampal region as well [21]. This suggests that this might be not an area-specific phenomenon, but rather a more general mechanism. Interestingly, a number of genes are regulated by stress in the hippocampal CA3 as well as in the PVN, e.g. DPYSL2, SNAP25, GNAO1 [21]. Like for the stress-regulated genes in CA3, we used also for the regulated genes in the PVN a pathway building program to propose novel signal transduction pathways elicited after stress. This pathway analysis revealed an interesting link between GNAi2 and APP whose stress regulation we validated by real time qPCR.

GNAi2, that displayed increased expression after stress, is a plasma membrane protein and a member of the G(i) proteins that inhibit adenylate cyclase. Many important hormones and neurotransmitters, including acetylcholine, dopamine and serotonin, use the G(i) pathway to evoke physiological responses [41]. In addition to inhibiting adenylate cyclase, G(i) regulates c-Src (and thereby STAT3) and Rap1 pathways, for example. This well studied inhibition may be physiologically relevant, in particular in inhibiting the effects of cAMP to modulate secretion in response to beta-adrenergic stimuli [42,43]. Despite their established role in modulating cellular processes, very little is known regarding molecular mechanisms underlying the transcriptional regulation of any G-protein. It has been reported previously, that the increase of reactive oxygen species in K562 cells up-regulates Galpha(i2) [44]. Here we report an increase of GNAi2 in DBA/2J mice 4 h after stress, which reveals a more direct impact of stress on GNAi2 expression by psychological stressors not reported so far.

Similarly to GNAi2, we were able to identify an increase of APP mRNA in the PVN of DBA/2J mice, 8 h after exposure to forced swimming. APP is mostly known for being the source of the toxic amyloid-β (Aβ) peptide found in neuritic plaques of Alzheimer's disease (AD) patients. However, it may in addition be a functionally important molecule in its full-length configuration, as well as be the source of numerous fragments with varying effects on neural function [45]. Besides its cellular function (axonal transport, cell adhesion, cholesterol metabolism and gene transcription) throughout the body, it is also reported to participate in a number of important functions in the CNS, i.e. neuronal development, survival and plasticity [46] and to be a central molecule in many metabolic and regulatory pathways, so that its regulation may impact on a network of genes [45]. Moreover, there is a growing body of evidence that APP may be part of the cellular response to stress, and serve neurotrophic and neuroprotective functions [47,48], although the neuroprotective role of APP remains controversial.

Our observed up-regulation of APP is in line with the rapid increase in amyloid precursor protein immunoreactivity in the supraoptic and paraventricular nuclei described in the rat hypothalamus after osmotic stress [49]. Furthermore, exposure of wild-type animals to an enriched environment can up-regulate APP-expression [50], although it remains unclear whether full-length APP or one of its fragments are involved Since APP has been reported to exert beneficial effects for neurons [48,51-53], the increase after stress might be important as a protective mechanism for the sensitive PVN area.

Linked to the action of APP, we also observed a regulation of the α-secretase ADAM10 8 h after stress. Upon neuronal over-expression in a mouse model for Alzheimer's disease, this protease has been shown to increase the secretion of sAPPα, reduce the formation of Aβ peptides and prevent their deposition in plaques as well as to alleviate impaired long-term potentiation and cognitive deficits [54]. In the stress exposed mice investigated here, there was pronounced strain specificity, i.e. 8 h after stress ADAM10 was strongly down-regulated in C57BL/6J mice (Table 3), but up-regulated in DBA/2J mice (Table 5). Of note, the inhibition of adenyl cyclase, from GNAi2 could also lead to the non amyloidogenic a-secretase pathway, resulting elevated sAPPα by likely shifting to the protein kinase competing signalling pathway. Moreover, this increase of GNAi2 after stress appears also to be strain-specific, because it was found in DBA/J2, but not in C57BL/6J mice [55,56]. The hypothesis emerging from these observations, i.e. a role of sAPPα in differentially shaping stress response needs to be tested by further experiments, for example by more directly manipulating the GNAi2 signalling pathway, ADAM10 expression and the activity and APP or its metabolite sAPPα, in vitro and possibly also in vivo. These studies could include stress-exposure and antidepressant response in APP transgenic and/or APP knockout mice.

The clustering analysis we have performed and the pathway analysis that followed turned out to be very useful tools and revealed new possible signalling pathways involving GNAi2 and APP. Obviously, mechanisms in addition to gene expression, such as protein phosphorylation, protein-protein binding etc. also operate in regulatory networks. Nevertheless, we believe that the identified network adds significantly towards the understanding of the complicated mechanisms of the response of the PVN to stress. Moreover, we propose that the combination of our results with future results expected from research efforts targeted towards proteins, gene polymorphisms, epigenomes, metabolome etc. will help identifying markers for diagnosis, stratification of subjects, and possibly also novel drug targets.

## Conclusions

Given the importance of PVN hypothalamic area for the physiological stress response and the discussed neuroprotective role of APP, the up-regulation of GNAi2 and APP mRNA levels after a mild stress in mice is suggested as it could be an adaptational stress-response in stress-responsive mice. Novel molecular pathways involving stress regulated genes that respond to stress in the PVN area, have been revealed based on clustering and signalling cascade pathway analysis.

## Methods

### Animal Experiments

Our study was performed with C57BL/6J and DBA/2J male mice with an age of 3-5 months (supplied from Harlan Winkelman, Borchen) and single housed under a 06:00-18:00 light cycle and subjected to forced swimming to induce an emotional stress, as previously described (Tsolakidou et al 2008). Briefly, the animals were placed in a 24 cm high 11 cm diameter cylinder filled with water at 22-25°C for 5 min. Afterwards they were transferred to their own original cage (i.e. single housed again). The animals elicited a mixture of behavioural activity that can be described as climbing, swimming and immobility, with the latter reflecting a passive stress coping behaviour. The experimental procedures were conducted in accordance with the Guide for the Care and Use of Laboratory Animals of the European Union (European Communities Council Directive 86/609/EEC) and approved by the Government of Bavaria, Germany.

The mice were separated into three groups per strain as previously described (Tsolakidou et al 2008) (see Fig. 1B). The first and the second group were subjected to stress at 8:00 or at 12:00, respectively. The third group was not stressed and further on used as reference group for the microarray experiments. All animals were decapitated at 16:00 to avoid possible interference by circadian variations of corticosterone (CORT) levels. Thus, the first and the second group have been actually sacrificed 8 h or 4 h after stress, respectively. Trunk blood was collected for determination of ACTH concentrations (10 animals/condition) and dissected brains from the same animals were frozen on dry ice and stored at -80°C (to be used for expression profiling). To monitor hormone levels acutely after stress, a small set of animals (5/condition) were sacrificed acutely at the respective time points (8:00 and 12:00). Plasma ACTH concentrations were determined in a radioimmune assay (ICN Biomedicals).

### Micropuncture and RNA preparation

Micropuncturing of the PVN and adjacent region on coronal tissue sections (200 μm thick) was applied under dry ice-cold conditions. To control for the accuracy of the puncture the sections were stained (cresyl violet) afterwards. Total RNA was extracted from the collected tissue (Trizol protocol, GibcoBRL). Samples from six animals were pooled to minimize the impact of biological variance, which is intrinsic to all organisms and can be substantial even in inbred mice [57]. After two rounds of amplification (Amino Allyl MessageAmp aRNA kit, Ambion) the RNA was labelled with Cy3 or Cy5 dyes (Amersham)

### Microarray hybridization and Analysis

Spotted cDNA microarray chips (MPIP 24K microarray platform with sequences from 19900 different genes, [58] were used, which allow a greater degree of flexibility in the choice of arrayed elements. Internal controls (sequences from other organisms) were used as contribution to ensure the quality of the data [59]. The microarray experiments were performed by competitive hybridisation of two differentially labelled (Cy3 or Cy5) probes of amplified total RNA samples. 10 arrays were used for each comparison, that is 5 technical replicates and a dye-swap with another 5 technical replicates. 20 μg of each Cy3- or Cy5-labeled sample were denatured at 95°C for 3 min in hybridisation buffer (50% formamide, 50 mM sodium phosphate-buffer pH 7.0, 5× Denhard's solution [Sigma, Taufkirchen, Germany], 6× SSC, 0.5% SDS, 0.4 mg/ml murine COT1-DNA [Invitrogen] and 5 μg poly(dA) [Amersham]). The hybridisation was performed in chambers submerged in a water bath at 42°C for 16 h. The arrays were washed for 15 min with 2× SSC/0.2% SDS at 60°C, in 0.5× SSC for 15 min at 60°C, rinsed in 0.2× SSC for 1 min at room temperature, shaken vigorously in 0.05× SSC at room temperature and finally air dried. All slides were scanned immediately afterwards.

Scanning was performed using a ScanArray 4000 laser scanner and ScanArray 3.1 Software (Perkin Elmer, Boston, USA) with a fixed PMT gain of 80%, and 98% (Cy3) or 70% (Cy5) laser power. The QuantArray software 2.1.0.0 (Perkin Elmer, Germany) and the fixed circle-analysis method were used to perform the quantification. Data were imported into a PostgreSQL relational database for further analysis. Raw data were normalized according to the procedure outlined elsewhere [60] and subjected to a two-sided one sample t-test for significantly differential expression. Candidate genes were screened for using thresholds of |fold regulation| 1.414 and |Z-score| 1.423. In order to exclude any possibility of wrong annotation the majority of the differentially expressed genes, in particular those of major interest were sequenced. All data are deposited at the GEO server (GSE20877).

### Ontology Groups Classification and Molecular Pathways Building

To classify and characterize the differentially expressed genes that resulted from the microarray analysis the Gene Ontology data annotation (version 161) (http://www.geneontology.org, http://www.ebi.ac.uk/GOA/) was used. In addition, the text mining program Pathway Studio 5.0 (Ariadne Genomics, Rockville, USA) was used for the identification of pathways linking the regulated genes. Downstream targets of the genes regulated 4 h after stress were looked for in the group of genes regulated 8 h after stress and upstream regulators of the latter in the former group. The main criteria applied for the final selection of the pathways were: 1. the last step of the pathway has to indicate molecular synthesis/expression so as to validate the expression change described on the microarray result, 2. confirmation of the annotation of the selected genes by sequencing and 3. validation of the literature references used by the program.

### *In situ *Hybridisation

18 μm thin cryostat sections prepared from frozen stored brains were thaw-mounted on poly L-Lysine-coated slides, dried and kept at -80°C. [α-^35^S] UTP-labelled riboprobes for amyloid β (A4) precursor protein (APP) and guanine nucleotide binding protein, alpha inhibiting 2 (GNAi2) genes were prepared by *in vitro *transcription from corresponding cDNA clones (sense probes were also included for control). The sections were dried, fixed in 4% paraformaldehyde, washed in PBS (3 times) and subjected to acetylation using 0.25% acetic anhydride in 0.1 M triethanolamine-HCL, pH 8.0. After subsequent dehydration in increasing concentrations of ethanol, brain sections were saturated with 100 μl of hybridisation buffer containing ~ 2.5 × 10^6 ^cpm ^35^S-labelled riboprobe. Brain sections were incubated overnight at 62°C or 58°C. Then, the sections were rinsed in 4× SSC (standard saline citrate), treated with RNAse A (20 mg/L) and washed in increasingly stringent SSC solutions at room temperature. Finally sections were washed in 0.1× SSC for 1 h at 67°C or 64°C (respectively) and dehydrated through increasing concentrations of ethanol. Autoradiography was on Biomax MR film (Eastman Kodak Co., NY, USA) for 3-5 days. The autoradiographs were digitised, and relative expression levels were determined by computer-assisted optical densitometry (Image J, Scion Corporation). The average value of 4-6 measurements was calculated from each animal [61].

### Quantitative (q)PCR

A total of 200 ng of amplified RNA from the first amplification round of the microarray analysis was reverse transcribed with Superscript II (Invitrogen, Karlsruhe, Germany) using random hexamer primers (Ambion) according to the manufacturer's protocol. For quality control, a small aliquot of each cDNA was analyzed on an agarose gel.

cDNA of DBA/2J and C57BL/6J mice from the non stressed, 4 h- and 8 h-after stress groups (for DBA/2J: N = 6 in each group; for C57BL/6J: N = 5 for non stressed and 8 h-after stress samples each, N = 6 for the 4 h stress group), was analyzed by qPCR, using the QuantiFast SYBR Green PCR Kit (Qiagen GmbH, Hilden, Germany) according to the manufacturer's instructions. The respective oligonucleotide primers were for *GNAi2 *(fwd: 5' CACCTCCATCATCCTCTTCC 3', rev: 5' GCACGTGAAGTGCGTGTAGA 3'), *APP *(fwd: 5' CGGAAGAGATCTCGGAAGTG 3', rev: 5' TGTTCGAACCCACATCTTCA 3') and the house keeping genes glyceraldehydes-3-phosphate dehydrogenase (GAPDH: fwd: 5' CCATCACCATCTTCCAGGAGCGAG 3', rev: 5' GATGGCATGGACTGTGGTCATGAG 3') hypoxanthine guanine phosphoribosyl transferase 1 (HPRT1; fwd: 5' GTCAAGGGCATA TCCAACAACAAAC 3', rev: 5' CCTGCTGGATTACATTAAAGCACTG 3') polymerase (RNA) II (DNA directed) polypeptide B (POLR2B; fwd: 5 'CAAGACAAGGATCATATCT GATGG 3', rev: 5' AGAGTTTAGACGACGCAGGTG 3') or ribosomal protein L13a (RPL13A; fwd: 5' CACTCTGGAGGAGAAACGGAAGG 3', rev: 5' GCAGGCATGAGGC AAACAGTC 3'). Experiments were performed in duplicates on a Lightcycler^®^2.0 instrument (Roche Diagnostics, Mannheim, Germany) under the following PCR conditions: Initial denaturation at 95°C for 10 min, followed by 40 cycles of denaturation (95°C for 10 s) and a combined annealing and extension phase (60°C for 30 s). At the end of each run, a melting curve (50-95°C with 0.1°C/s) was recorded to ensure the quality of the PCR product. Crossing points (Cp) were calculated by the LightCycler^®^Software 4.0 (Roche Diagnostics GmbH) using the absolute quantification fit points' method. Threshold and noise band were set to the same level in all runs used in a comparison. Relative gene expression was determined by the 2^-ΔΔCT ^method [62] using the real PCR efficiency calculated from an external standard curve. Cp were normalized to the housekeeping genes GAPDH, HPRT1, POLR2B or RPL13A). Fold regulation values were calculated relative to the expression mean of basal mice. The calculation Cp (mean (GAPDH, HPRT1, POLR2B or RPL13A)) - Cp (mean (cand. gene)) was used for each animal.

RT-qPCR experiments were analyzed using non-parametric tests (Mann Whitney using SPSS 12.0).

### Identifying genes with similar regulation profiles/Clustering Analysis

To identify genes that show similar expression ratios across time points, i.e. genes that might be co-regulated or affecting each other in a common pathway, we used cluster analysis. Cluster analysis allows the grouping of expression profiles with respect to their relative similarity or, in mathematical terms, a "distance". We consider expression profiles (the expression ratios of the different measurements) to be similar--and thus having a small distance--when they fulfil two criteria: 1. show a high absolute (not mean-corrected) correlation, and 2. have either the same or the opposite regulation (up or down) at all corresponding measurements.

Translated into distance between expression profiles this means that expression profiles, that can be scaled (with either a positive or negative factor, but not shifting the zero line) onto each other, have a small distance. This distance measure groups genes with similar regulation patterns as close neighbours in the cluster analysis. For APP and GNAi2 we show the respective neighbourhoods (targeting at least 10 neighbouring genes, but showing all genes in the most distant branch) as depicted by the corresponding dendrograms.

## Authors' contributions

AT performed the animal experiments (including hormone measurements), micropuncture, RNA preparation, microarray, Ontology Groups Classification, *in situ *hybridisation, molecular pathways building, interpretation of data, and manuscript writing. LC performed the real time PCR experiments, BP the microarray analysis as well as the clustering analysis, and DT the PathwayStudio analyses. MP contributed to microarray analyses and ontology group classification. JMD supervised microarray experiments, WW PathwayStudio analyses. IS contributed to project design, RL to real time PCR supervision, and FH to shaping the project concept. TR designed the project and wrote the manuscript together with AT.

All the authors have read and approved the final manuscript.

## Supplementary Material

Additional file 1 **Supplemental Table 1**. Genes with more than 1.4 fold expression differences between the two mouse strains (+: higher expressed in DBA/2J vs C57BL/6J and -: lower expressed in DBA/2J vs C57BL/6J). Click here for file

Additional file 2 **Supplemental Table 2**. Genes that are more than 1.4 fold up- or down-regulated 4 h after stress in C57BL/6J. Click here for file

Additional file 3 **Supplementary Table 3**. Genes that are more than 1.4 fold up- or down-regulated 8 h after stress in C57BL/6J. Click here for file

Additional file 4 **Supplementary Table 4**. Genes that are more than 1.4 fold up- or down-regulated 4 h after stress in DBA/2J mice. Click here for file

Additional file 5 **Supplementary Table 5**. Genes that are more than 1.4 fold up- or down-regulated 8 h after stress in DBA/2J mice. Click here for file

Additional file 6 **Supplemental Figure**. Graph of the expression profiles of genes with similar expression changes revealed by cluster analysis when considering both up- and down-regulation. Click here for file
